# Sensory Loss and Delirium Among Medicare Beneficiaries

**DOI:** 10.1093/geroni/igaa057.2890

**Published:** 2020-12-16

**Authors:** Emmanuel Garcia Morales, Nicholas Reed

**Affiliations:** 1 Johns Hopkins Bloomberg School of Public Health, Baltimore, Maryland, United States; 2 Johns Hopkins University, Baltimore, Maryland, United States

## Abstract

Sensory impairment is prevalent among older adults and may increase risk for delirium via mechanisms including sensory deprivation and poor communication which may result in confusion and agitation. In the Medicare Current Beneficiary Study (MCBS), delirium was measured using a validated algorithm of claims data. Sensory impairment was defined as any self-reported trouble hearing or seeing, with the use of aids, and was categorized as no impairment, hearing impairment only (HI), vision impairment only (VI), and dual sensory impairment (DSI). Among, 3,240 hospitalized participants in 2016-2017, 346 (10.7%) experienced delirium. In a model adjusted for socio-demographic and health characteristics, those with HI only, VI only, and DSI had 0.84 (95% CI: 0.6-1.3), 1.1 (95% CI 0.7-1.7), and 1.5 (95% CI 1.0-2.1) times the odds of experiencing delirium compared to those without sensory impairment. Future research should focus on mechanisms underlying association and determine the impact of treatment of sensory loss.

